# Chloroplast genome characterization of *Rubus arcticus* L.

**DOI:** 10.1080/23802359.2022.2130715

**Published:** 2022-10-18

**Authors:** Zhehui Jiang, Tianhao Wang, Yuan Gao, Dingxi Shu, Xiangquan Li, Weichao Ren, Wei Ma, Yihong Bao

**Affiliations:** a School of Forestry, Northeast Forestry University, Harbin, China; b College of Mechanical and Electrical Engineering, Northeast Forestry University, Harbin, China; c College of Chemistry, Chemical Engineering and Resource Utilization, Northeast Forestry University, Harbin, China; d Bila River National Nature Reserve Authority, Oroqen Autonomous Banner, Hulunbuir City, China; e Yichun Branch of Heilongjiang Academy of Forestry, Yichun, China; f Pharmacy College, Heilongjiang University of Chinese Medicine, Harbin, China

**Keywords:** *Rubus arcticus*, Rosaceae, chloroplast genome, species identification

## Abstract

*Rubus arcticus* Linnaeus (1753) is a medicinal and edible plant in the Rosaceae with wide distribution in northeast China. The total length of the genome was 156,668 bp with a GC content of 37.1%, including a large single-copy (LSC, 85,958 bp) region, a small single-copy region (SSC, 18,756 bp), and inverted repeat (IR, 51,954 bp) regions. A total of 129 genes were identified. The numbers of protein genes tRNAs and rRNAs were 85, 36, and 8, respectively. Phylogenetic analysis indicated that *R. arcticus* belongs to the *Rubus* genus. Published *R. arcticus* chloroplast genomes have yielded insights into the closely related species identification, phylogenetic position and *Rubus* evolution.

*Rubus arcticus* Linnaeus (1753) is a rootstock semi-subshrub possessing an optional root sucker with unspecialized late morphological disintegration (Gudovskikh et al. 2021). It is a Eurasian-North American arctic-boreal species that is mainly spread in the temperate zone of northern regions. Wild *R. arcticus* produces berries that contain a variety of nutrients and have high economic value. Previous studies have focused on the relationship between fluctuations in yield and the influence of weather conditions on *R. arcticus* using modified droplet-vitrification cryopreservation (Kostamo et al. 2018). Russian researchers have investigated the distribution of this species in the Kirov region of Russia. Currently, 10 cultivars of *R. arcticus* are clearly distinguished based on AFLP marker data at the molecular level (Lindqvist-Kreuze et al. 2003). Additionally, the chloroplast genome of other *Rubus* species has been obtained, while the chloroplast genome of *R. arcticus* has not been reported (Yang et al. 2017; Zhu et al. 2019; Liu et al. 2021; Zhang et al. 2021). Therefore, the chloroplast genome of *R. arcticus* was sequenced for the first time using a second-generation high-throughput sequencing platform.

Fresh leaves of *R. arcticus* were collected from Heilongjiang Province, China (N 48°28′53.25″, E129°20′54.25″); the leaves were dried in silica at the collection site and stored at −80 °C in the laboratory. The voucher herbarium specimen (YCL20210620002) was stored at Northeast Forestry University, Harbin City, Heilongjiang Province (https://forestry.nefu.edu.cn/, Zhehui Jiang, zhehuijiang@126.com). Total genomic DNA was extracted using a Plant Genomic DNA Kit (SIMGEN, Hangzhou, China). Genome sequences were obtained using an Illumina NovaSeq 6000 (Illumina, San Diego, CA) with a paired-end library. The 5.42 G of raw data were collected by us, and the 5.4 G of clean data were filtered with fastp software. The chloroplast genome was *de novo* assembled through GetOrganelle (V1.7.5) (Jin et al. 2020). The CPGAVAS2 (Shi et al. 2019) online software was employed to annotate the chloroplast genome, with *R. xanthoneurus* as a reference. The chloroplast genome sequence was submitted to NCBI (GenBank: OL891648).

The chloroplast genome of *R. arcticus* was 156,668 bp in length. It had a typical quadripartite structure with a large single-copy (LSC) region, a small single-copy region (SSC), and two inverted repeat regions (IRa and IRb). The lengths were 85,958 bp, 18,756 bp, and 25,977 bp, respectively. A total of 129 genes were annotated, including 85 protein-coding genes, 36 tRNAs, and eight rRNAs. The overall GC content of the chloroplast genome was 37.1%.

Phylogenetic analysis is essential for species identification and phylogenetic evolution. In the current work, the chloroplast genome of *R. arcticus*, 27 other species and a variant of the *Rubus* genus were subjected to phylogenetic analysis with three species of *Rosa* genus as the outgroup. The chloroplast genome of all the collected species was aligned by MAFFT (v7.481) (Katoh et al. 2019), and the best model for phylogenetic analysis was identified using PhyloSuite software (Zhang et al. 2020). We constructed an ML tree with IQtree (v2.1.3) (Minh et al. 2020) based on the TVM + F+I + G4 model with 1000 bootstraps (Figure 1). According to the results, all species of *Rubus* genus were clustered together into one clade, and *R. arcticus* as a species of Sect. *Cylactis* forms a monophyletic branch.

**Figure 1.**
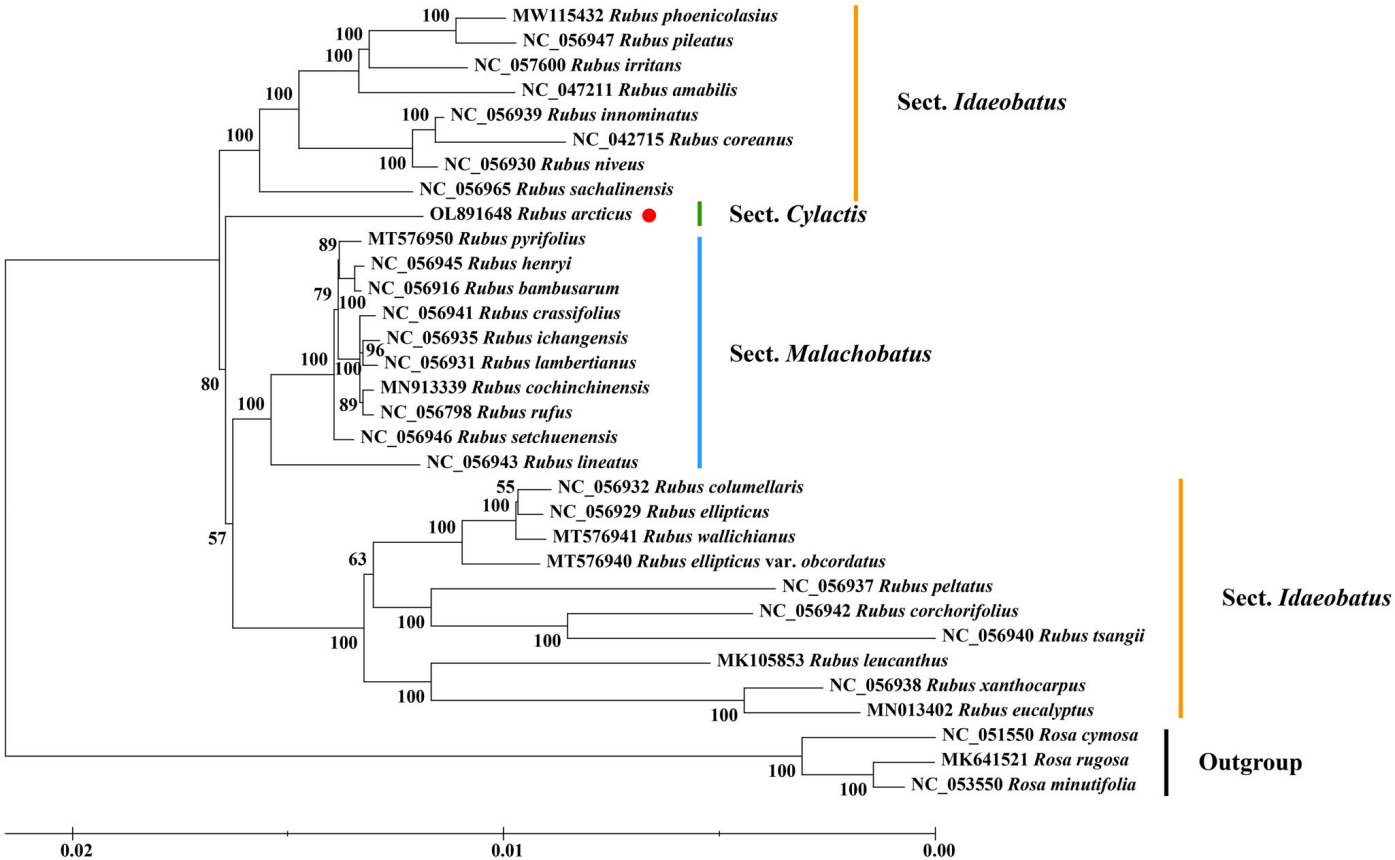
Phylogenetic analysis of 29 *Rubus* species based on maximum-likelihood with 1000 bootstraps and three species of *Rosa* genus as outgroup.

## Data Availability

The data that support the findings of this study are openly available in GenBank (https://www.ncbi.nlm.nih.gov/) under accession number of OL891648. The associated BioProject, SRA, and Bio-Sample numbers are PRJNA789688, SRR17253322, and SAMN24146691, respectively.
